# Experimental Demonstration of Spectral Intensity Optical Coherence Tomography

**DOI:** 10.1038/srep22126

**Published:** 2016-02-26

**Authors:** Piotr Ryczkowski, Jari Turunen, Ari T. Friberg, Goëry Genty

**Affiliations:** 1 Optics Laboratory, Department of Physics, Tampere University of Technology, 33720 Tampere, Finland; 2 Institute of Photonics, University of Eastern Finland, P. O. Box 111, FI-80101 Joensuu, Finland

## Abstract

We demonstrate experimentally spectral-domain intensity optical coherence tomography using a Mach-Zehnder interferometer with balanced detection. We show that the technique allows for a point spread function with reduced full-width at half maximum compared to conventional optical coherence tomography. The method further provides benefits similar to those of chirped-pulse interferometry in terms of dispersion cancellation but only requires a broadband incoherent source and standard detectors. The measurements are in excellent agreement with the theoretical predictions. Finally, we propose an approach that enables the elimination of potential artefacts arising from multiple interfaces.

Optical coherence tomography (OCT) is a powerful, three-dimensional (3D) imaging technique which may be operated in both the spectral and the temporal domain^1^,^2^, and it is widely employed for biological *in vitro* and *in vivo* imaging^3^. The axial resolution of conventional OCT is limited by the spectral bandwidth of the light source and by the dispersion of the optical components and/or the sample under the test. Because of dispersion, especially in fiber-based setups, increasing the spectral bandwidth of the light source may not necessarily lead to resolution improvement^4^. To circumvent this problem several approaches have been proposed, including both numerical^5^,^6^ and experimental techniques^7^,^8^,^9^,^10^,^11^,^12^,^13^. In particular, methods such as quantum-optical coherence tomography (QOCT) or chirped pulse interferometry have been shown to possess built-in dispersion cancelation and resolution enhancement; however, these techniques generally require sophisticated light sources such as single-photon sources or chirped ultrashort pulses and advanced detection techniques^9^,^10^,^11^,^12^,^13^. They also operate in the time domain requiring in-depth scanning, which results in slow measurement times.

Intensity-based optical coherence tomography, based on higher-order correlations compared to standard OCT, using a classical broadband light source and a Mach-Zehnder interferometer with balanced detection was recently put forward in the time domain^14^,^15^. The method produces improved resolution but is hampered by the lack of ultrafast detectors capable of recording rapid intensity variations characteristic of incoherent sources. Spectral intensity optical coherence tomography (SIOCT) on the other hand provides a much simpler and cost-effective alternative that operates, without moving parts, in the spectral domain and can use a classical broadband light source of any state of temporal coherence and standard detectors^16^,^17^. Furthermore, SIOCT requires only a minor modification of conventional spectral-domain OCT imaging setup, exhibits a point spread function (PSF) with reduced full-width at half maximum compared to standard OCT, and, significantly, possesses built-in even-order dispersion cancellation. Yet, no experimental demonstration of SIOCT has been reported. Numerical techniques based on higher-order correlations that yields the frequency-depth distribution^18^ have been suggested and demonstrated^19^ to provide useful information when the spectrum of backscattered light are depth-varying along the imaging axis. These techniques require the use of multiple windows to maintain high resolution in frequency and depth simultaneously. Here, we demonstrate experimentally SIOCT with a classical incoherent source, confirming both the reduced PSF and the dispersion cancellation in the case of a single interface. We also measure a sample with two interfaces and show how simple numerical post-processing similar to that used to obtain the Wigner distribution of time-varying frequency fields^18^ allows to discriminate artefacts arising from cross talk in the higher-order correlation signal. The measurements are in excellent agreement with the theoretical predictions. Our results open up new perspectives for high-resolution imaging using classical light sources.

## Theory

**Figure 1:**
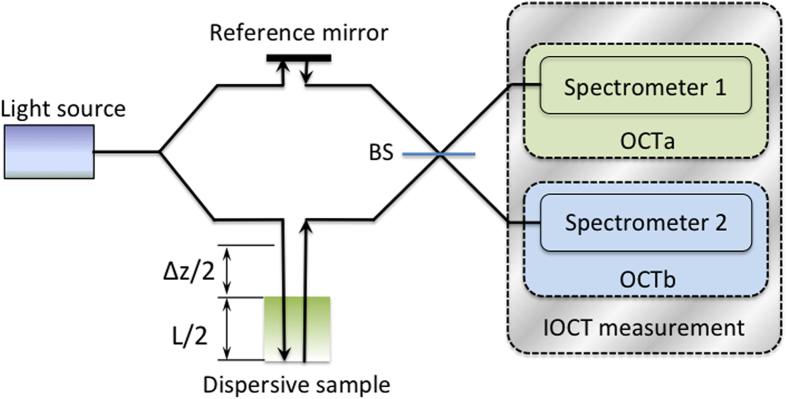
OCT and SIOCT concepts. BS – 50:50 non-polarizing beam splitter. OCT corresponds to a single spectral interference measurement at Spectrometer 1 (or 2). SIOCT corresponds to a simultaneous measurement of spectral interferences at Spectrometers 1 and 2.

The difference between the principles of the operation of conventional spectral-domain OCT and SIOCT is illustrated in Fig. 1. Light from an incoherent broadband source is divided between the two arms of an interferometer. One arm serves as the reference while the other contains the sample to be characterized. In traditional OCT, the spectral intensity recorded at the output of the beam splitter and resulting from the interference of the light in the two arms is of the form


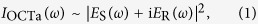



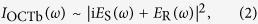


where *ω* is the angular frequency, *E*_S_ and *E*_R_ are the complex spectral amplitudes of the electric fields emerging from the sample and reference arms, respectively, and the subscripts a and b denote at which output of the beam splitter the spectral OCT measurement is recorded. The phase difference between the reference and sample arm fields is caused by the difference Δ*z* in distance that the light propagates in air and the propagation through the dispersive sample of path length *L*. Both distances mark the total path, including wave transits before and after reflection. The intensity of the recorded interferogram can then be written as


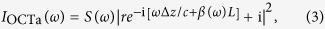


where *S*(*ω*) represents the spectrum of the light source, 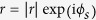 is the (complex) amplitude reflection coefficient of the sample, and *c* is the speed of light in vacuum. The coefficient *β*(*ω*) denotes the frequency-dependent propagation constant within the sample. Using a Taylor-series expansion around the central frequency *ω*_0_ of the source spectrum up to second order, one obtains


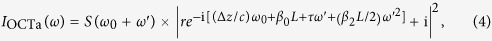


where 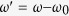 and 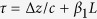, with *β*_0_, *β*_1_, and *β*_2_ being the propagation constant, the group delay, and the group-velocity dispersion at frequency *ω*_0_, respectively. The envelope of the spectral interference pattern represents the source spectrum whilst the frequency, phase, and amplitude of the modulation underneath depend on the sample position and reflectance, which can then be obtained through a Fourier transform of the recorded interferogram. The full width at half maximum (FWHM) of the PSF is given by the Fourier transform of the term corresponding to the optical path difference between the reference and sample arms. In the absence of dispersion, the PSF is inversely proportional to the source bandwidth. With dispersion present in the system, the resolution decreases by a factor 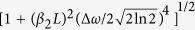, where Δ*ω* is the FWHM spectral bandwidth of the light source.

In SIOCT, the spectral interference patterns of the fields in the two arms are recorded simultaneously by two separate detectors at the two output ports of the beam splitter. The location and reflectance of the sample are obtained from the Fourier transform of the cross product of the individual spectral intensities 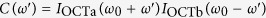. It is straightforward to show that the interference pattern of the SIOCT signal is then given by


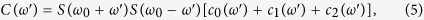


where


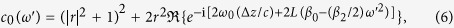



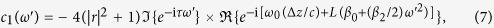



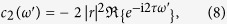


with ℜ and ℑ denoting the real and the imaginary parts, respectively. One can see that the Fourier transform of the SIOCT signal produces a PSF with a width reduced by a factor of 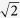 compared to that of conventional OCT. The Fourier spectrum of the function *C*(*ω*′) gives access to the sample information and consists of three separated peaks corresponding to the Fourier transforms of 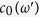, *c*_1_(*ω*′), and *c*_2_(*ω*′) convolved by the Fourier transform of 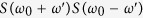.

Several observations can be made. We first remark that the product 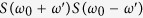 is always an even function, even if the spectrum of the light source is not symmetrical with respect to *ω*_0_. The term 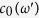 is an even function whose Fourier transform produces a real-valued peak centered at the zero delay (equal path lengths). The term 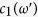, on the other hand, is an odd function that depends on the optical path length and its Fourier transform corresponds to an imaginary-valued peak centered at the optical path difference between the two arms. Finally, the Fourier transform of 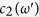 gives rise to a real, negative-valued peak at twice the optical path difference. It is precisely this peak in the Fourier spectrum that gives information about the sample position and, after correcting for the distance, one obtains an overall PSF reduced by a factor of 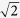 compared to standard OCT. However, it should be noted that this decrease in the PSF width comes with a price: the imaging depth is only half of that obtained by the standard OCT. We further note that the number of data points is identical in both OCT and SIOCT but their density is doubled in SIOCT, allowing for better accuracy in locating interfaces. Note further that in practice the terms arising from 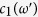 and 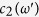 can be distinguished as one is real and the other imaginary. Besides the point density increase in the measurement, another benefit of SIOCT is the inherent dispersion cancellation of all even-order terms in the Taylor-series expansion. This is because in SIOCT intensities of opposite frequencies relative to central frequency *ω*_0_ are multiplied, cancelling the even-order phase terms that arise from dispersion.

In the case of multiple interfaces, artefacts which have generally complex values appear in the Fourier signal due to cross-talk between the terms corresponding to the multiple interferences. This means that they can not be discriminated from the real interfaces by solely considering the real part of the Fourier transform of the SIOCT interferogram. Yet, in this case, one can take advantage of the fact that the amplitude of the artefacts corresponding to the 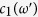 term in Eq. (7) depends on the center frequency *ω*_0_ of the source spectrum. Indeed, for a stationary light source the spectrum does not need to be symmetrical with respect to *ω*_0_, so that one can choose arbitrarily the center frequency as long as the detected signal significantly exceeds the measurement noise. Because the phase of the 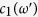 term oscillates with *ω*_0_, the amplitude of the peak in the Fourier domain corresponding to artefacts oscillates when spectra of different center frequencies are measured. This is illustrated in Fig. 3 where we show numerical simulations of the Fourier signal recorded for two interfaces separated by 95 *μ*m (optical thickness) as a function of the light source center wavelength 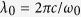. We show results both without [Fig. 3(a,b)] and with dispersion [Fig. 3(c,d)] present in the system. We can see how in both cases the peaks corresponding to the interface positions always remain negative with a change in amplitude that follows the spectral amplitude of the source vs wavelength. On the contrary, the amplitude of the peak corresponding to the artefact arising from cross-talk oscillates dramatically between negative and positive values. We also note that when dispersion is present, it is cancelled independently of the center wavelength with a constant width for the PSF. Interestingly, multiple measurements with a different center frequency can be numerically performed by post-selection of a smaller number of points from the measured interferogram, which in turn shifts the spectral window together with the central frequency. In this way, one can produce an ensemble of data sets corresponding to spectra with different central frequencies. Therefore, in principle, only a simple post-processing operation of the recorded interferograms is required to discriminate possible artefacts when multiple interfaces are probed.

## Experimental Results

**Figure 2:**
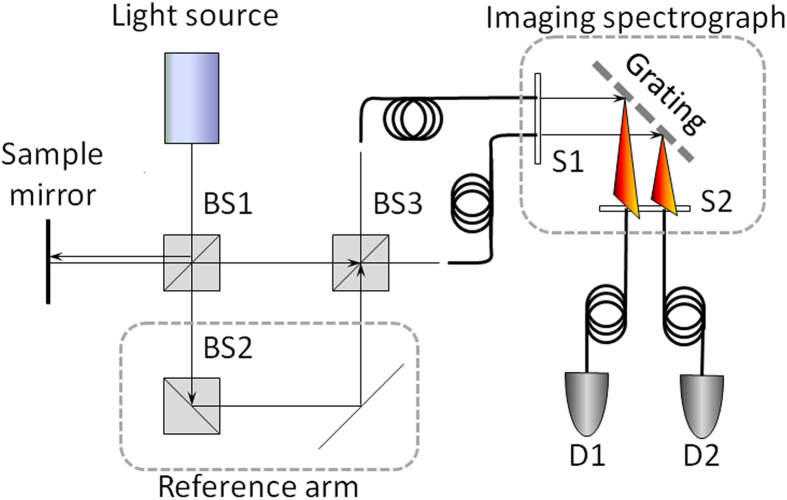
Modified Mach-Zehnder interferometer, experimental setup: BS – 50:50 non-polarizing beam splitter. S1, S2 – spectrograph input and output slits. D1, D2 – photodiodes.

We confirmed experimentally the narrowing of the PSF and dispersion cancellation in SIOCT as compared to standard OCT using a modified Mach-Zehnder interferometer with balanced detection. The experimental setup is shown in Fig. 2 (see also Methods). The signal recorded at either detector independently is identical to a conventional OCT system, while measuring simultaneously the signal at both detectors allows to construct the SIOCT interferogram.

In order to characterize the PSF, we first performed measurements in the absence of dispersion in the sample arm. The results are shown in Fig. 4 where both the real and the imaginary part of the Fourier transform of the cross-product function 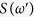 are presented. For comparison, the theoretical results obtained on the basis of Eqs. (4) and (5) are superimposed as circles. The optical path delay was converted into physical distance as light would travel in vacuum and thereby the 0 point represents equal path lengths. For ease of comparison with the conventional OCT result, the distance was divided by 2 for the SIOCT measurement so that the image peak would correspond to the actual sample position. In general, we observe excellent agreement between the experimental and theoretically predicted results. Specifically, we see that the measured imaginary part of the Fourier spectrum is an odd function and corresponds precisely to the Fourier transform of the term 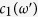, while the measured real part is even and matches closely the Fourier transforms of the terms 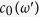 and 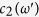 of the SIOCT interferogram. Significantly, we also observe how the width of PSF is reduced in comparison to that of standard OCT (see inset in Fig. 4). The FWHMs of the OCT and SIOCT PSFs corresponding to the sample position are respectively equal to 23.5 *μ*m and 17.3 *μ*m, giving a ratio of 0.736 close to the 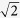 theoretical PSF width reduction predicted for a light source with a Gaussian spectrum.

We next proceeded to confirm experimentally the built-in dispersion cancellation of the SIOCT scheme. For this purpose, an 8 cm thick bulk piece of SF10 glass was inserted into the sample arm. In order to increase the effect of dispersion the beam was propagated eight times through the glass cube, effectively corresponding to a 64 cm glass piece. The group-velocity dispersion coefficient of SF10 at 1610 nm is 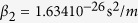, which should lead to a PSF width increase by a factor of about 2.4 in the case of conventional OCT. The measurement results for conventional OCT and SIOCT are illustrated in Fig. 5. As for the dispersionless case, the theoretical results computed from Eqs. (4) and (5) are also superimposed as circles. It can readily be seen that the experimental results in both cases follow the theoretical predictions with great accuracy. More specifically, we observe how in the case of conventional OCT the FWHM of the PSF corresponding to the sample location has broadened nearly 2.5 times to 58.0 *μ*m due to the dispersion of the SF10 glass, a value very close to that expected from the theoretical calculations. Most remarkably, the width of the SIOCT PSF at the sample position is unaffected by the presence of the bulk piece of glass, clearly demonstrating that dispersion is inherently canceled in SIOCT. In fact, the width of the PSF was found to be 16.2 *μ*m, which is even slightly reduced compared to the case when dispersion. This slight change in the resolution is caused by third-order dispersion, which is not canceled in SIOCT (only even order are). Finally, we also see how the artefact peak corresponding to the term 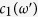 is generally affected by dispersion, which provides an additional means of discrimination.

**Figure 3:**
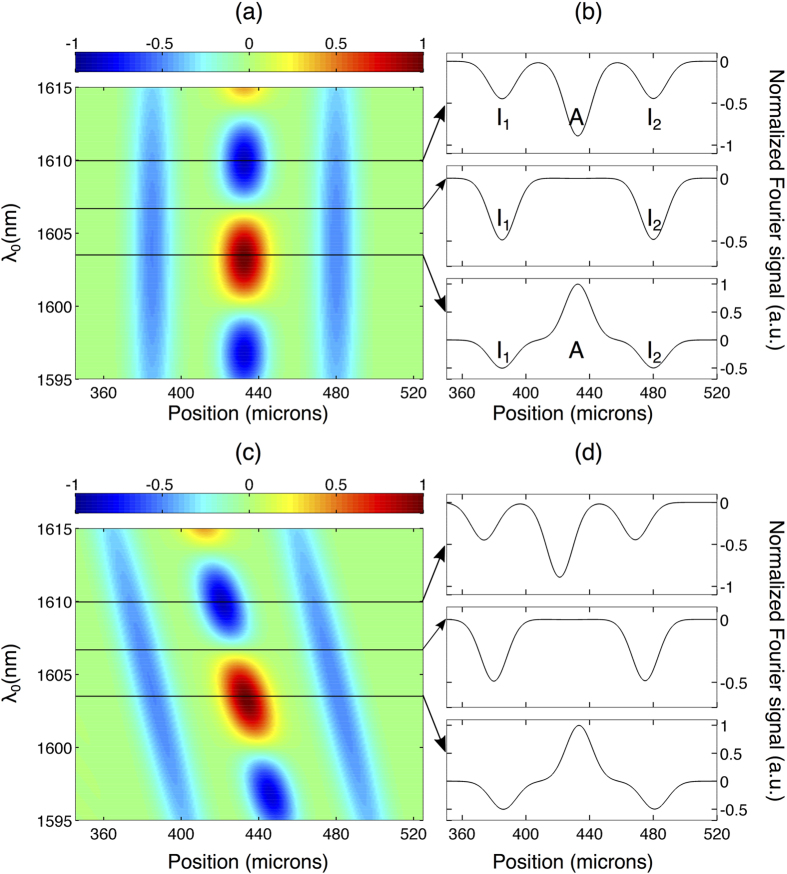
False color representation of the numerically simulated real part of the Fourier signal recorded by the IOCT system vs. center wavelength *λ*_0_ of the light source for two interfaces: (**a**) without dispersion and (**c**) with dispersion. Fourier signal for selected center wavelengths indicated by the arrows: (**b**) without dispersion and (**d**) with dispersion. For clarity, the position of the two interfaces (I_1_ and I_2_) and artefact A are marked.

**Figure 4:**
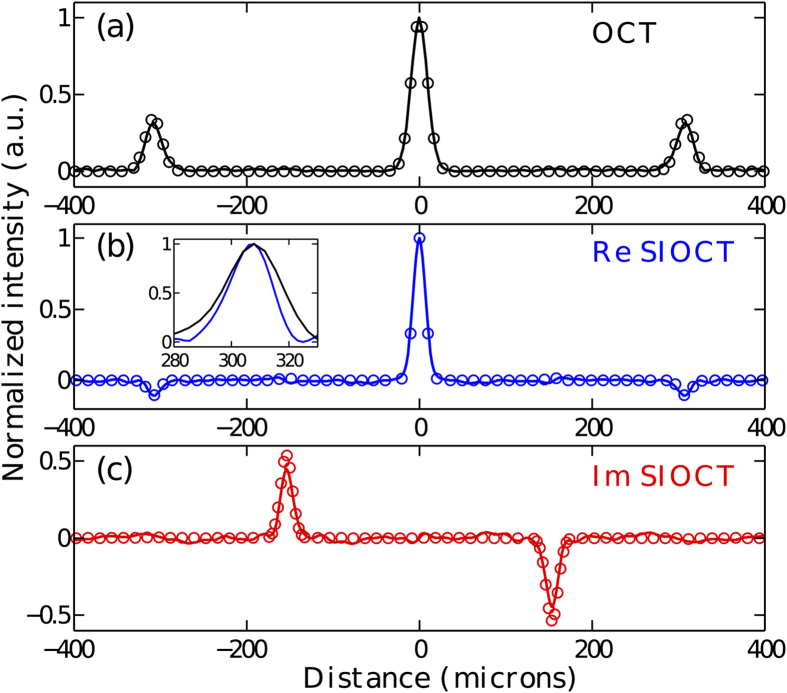
Comparison of OCT and intensity-based SIOCT in the absence of dispersion in the system. The solid lines show the experimental results and the circles correspond to theoretical predictions. (**a**) OCT result. (**b**,**c**) Real and imaginary part of the Fourier transform of the SIOCT interferogram, respectively. The inset in (**b**) displays a direct comparison of the PSF of both techniques: black OCT, blue SIOCT.

**Figure 5:**
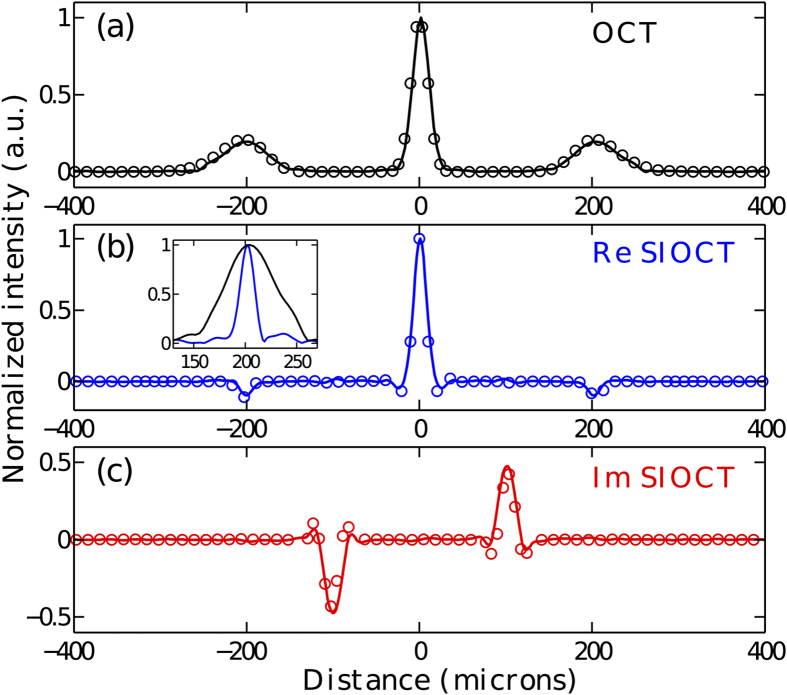
Comparison of OCT and intensity-based SIOCT when dispersion is present in the system. The solid lines show the experimental results and the circles give the theoretical predictions that are based on Eqs. (4) and (5). (**a**) OCT result. (**b**,**c**) Real and imaginary part of the Fourier transform of the SIOCT interferogram, respectively. The inset in (**b**) illustrates the direct comparison of the PSF in the two techniques: black OCT, blue SIOCT.

**Figure 6:**
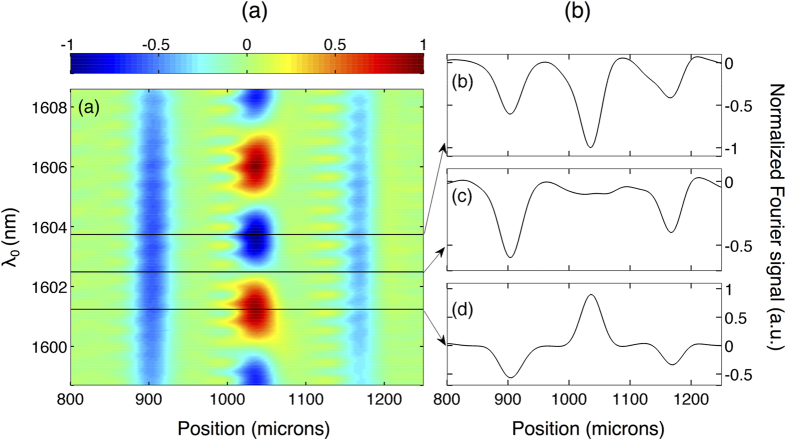
Experimental results for two interfaces. (**a**) False color representation of the numerically post-processed real part of the Fourier signal recorded by the IOCT system vs. center wavelength *λ*_0_ of the spectral window. (**b**) Fourier signal for selected center wavelengths as indicated by the arrows.

We also performed experiments with a dual-interface sample. for this purpose, we inserted a thin microscope cover slip consisting of two air-glass interfaces in the sample arm. Note that no dispersion was added in this case. The results are illustrated in Fig. 6. In order to discriminate the actual interfaces from the artefacts we processed the experimental data by post-selecting a smaller number of points from the measured interferograms which allows to shift numerically the spectral window and center frequency (or wavelength). A false color representation of the real part of the Fourier signal is shown in Fig. 6 as a function of the center wavelength. The amplitude of the artefacts oscillates between positive and negative values as the central wavelength is changed in perfect agreement with the numerical simulations of Fig. 3. In marked contrast, the amplitude of the peaks corresponding to the actual interfaces remains negative independently of the center wavelength of the source and their amplitude follow the spectral intensity of the source vs wavelength. The optical distance between the two interfaces can then be readily inferred to be c.a. 270 *μ*m, resulting in a measured physical thickness of 185 *μ*m for the cover slip in very good agreement with the manufacturer specified value of <200 *μ*m. This experimental result demonstrates a convenient approach to effectively eliminate artefacts in the case of samples with multiple interfaces.

## Conclusions

In summary, we have experimentally demonstrated spectral intensity optical coherence tomography and proved the associated reduced point spread function and dispersion cancellation. The imaging depth for SIOCT is only half of the one obtained by the standard OCT but the density of measurement points is doubled in SIOCT allowing for better accuracy in locating interfaces. We further suggested a possible approach to eliminate artefacts that might be present in the case of samples with multiple distinct interfaces. No fast detectors are needed in our technique as only mean spectral intensities are measured. The method works with classical light sources and standard photodiodes, illustrating the simplicity of the technique and showing its potential application to high resolution imaging.

## Methods

The light source used in the experiments was a fiber-coupled superluminescent diode (Exalos ESL 1620) with a center wavelength of 1604 nm. The source spectrum is close to Gaussian with a spectral bandwidth of 55 nm (FWHM). The light emitted by the source was collimated with a parabolic mirror and three identical non-polarizing beam-splitter cubes were used to divide the light among the sample and reference arms. A single, partially reflecting mirror placed on a manual translation stage to adjust the optical path length was used as the sample. The reference wave transmitted through beam splitter BS1 was reflected from an additional beam splitter BS2 in order to equalize the dispersion and phase shifts experienced by both beams due to the various optical elements. As a result, any difference between the complex amplitudes of the electric fields in the sample and reference arms before interfering on beam splitter BS3 is caused only by the path difference between the two arms and sample presence. Light at the two output ports of BS3 was coupled to a single-mode fiber using achromatic lenses. The fiber outputs were then placed on top of each other in the object plane of a monochromator (Horiba iHR 550) allowing to measure simultaneously the spectral intensities at the two detectors. On passing through the output slit of the monochromator, light was collected by multimode fibers and intensities were measured by InGaAs amplified diodes (Thorlabs PDA10CS). Lock-in detection was used to improve the signal-to-noise ratio. Wavelength scanning and data acquisition were controlled by a PC.

## Additional Information

**How to cite this article**: Ryczkowski, P. *et al.* Experimental Demonstration of Spectral Intensity Optical Coherence Tomography. *Sci. Rep.* **6**, 22126; doi: 10.1038/srep22126 (2016).

## References

[b1] Leitgeb R., Hitzenberger C. K. & Fercher A. F. Performance of fourier domain vs. time domain optical coherence tomography. Opt. Express 11, 889 (2003). 19461802 10.1364/oe.11.000889

[b2] de Boer J. F., Cense B., Park B. H., Pierce M. C., Tearney G. J. & Bouma B. E. Improved signal-to-noise ratio in spectral-domain compared with time-domain optical coherence tomography. Opt. Lett. 28, 2067 (2003). 14587817 10.1364/ol.28.002067

[b3] Fercher A. F., Drexler W., Hitzenberger C. K. & Lasser T. Optical coherence tomography - principles and applications. Rep. Prog. Phys. 66, 239 (2003).

[b4] Wojtkowski M. *et al.* Ultrahigh-resolution, high-speed, fourier domain optical coherence tomography and methods for dispersion compensation. Opt. Express 12, 2404 (2004). 19475077 10.1364/opex.12.002404

[b5] Fercher F. *et al.* Numerical dispersion compensation for partial coherence interferometry and optical coherence tomography. Opt. Express 9, 610 (2001). 19424297 10.1364/oe.9.000610

[b6] Banaszek K., Radunsky A. & Walmsley I. Blind dispersion compensation for optical coherence tomography. Opt. Commun. 269, 152 (2007).

[b7] Erkmen B. I. & Shapiro J. H. Phase-conjugate optical coherence tomography. Phys. Rev. A 74, 041601(R) (2006).

[b8] Gouët J. L., Venkatraman D., Wong F. N. C. & Shapiro J. H. Experimental realization of phase-conjugate optical coherence tomography. Opt. Lett. 35, 1001 (2010). 20364197 10.1364/OL.35.001001

[b9] Lavoie J., Kaltenbaek R. & Resch K. J. Quantum-optical coherence tomography with classical light. Opt. Express 17, 3818 (2009). 19259223 10.1364/oe.17.003818

[b10] Mazurek M. D., Schreiter K. M., Prevedel R., Kaltenbaek R. & Resch K. J. Dispersion-cancelled biological imaging with quantum-inspired interferometry. Sci. Rep. 3, 1582 (2013). 23545597 10.1038/srep01582 PMC3613801

[b11] Abouraddy A. F., Nasr M. B., Saleh B. E. A., Sergienko A. V. & Teich M. C. Quantum-optical coherence tomography with dispersion cancellation. Phys. Rev. A 65, 053817 (2002).

[b12] Nasr M. B., Saleh B. E. A., Sergienko A. V. & Teich M. C. Demonstration of dispersion-canceled quantum-optical coherence tomography. Phys. Rev. Lett. 91, 083601 (2003). 14525237 10.1103/PhysRevLett.91.083601

[b13] Nasr M. B., Saleh B. E. A., Sergienko A. V. & Teich M. C. Dispersion-cancelled and dispersion-sensitive quantum optical coherence tomography. Opt. Express 12, 1353 (2004). 19474956 10.1364/opex.12.001353

[b14] Lajunen H., Torres-Company V., Lancis J. & Friberg A. T. Resolution-enhanced optical coherence tomography based on classical intensity interferometry. J. Opt. Soc. Am. A 26, 1049 (2009). 10.1364/josaa.26.001049 19340281

[b15] Zerom P., Piredda G., Boyd R. W. & Shapiro J. H. Optical coherence tomography based on intensity correlations of quasi-thermal light. In *Conference on Lasers and Electro-Optics/Quantum Electronics and Laser Science Conference (CLEO/QELS), Baltimore, MD,* JWA48 (2009).

[b16] Shirai T. & Friberg A. T. Resolution improvement in spectral-domain optical coherence tomography based on classical intensity correlations. Opt. Lett. 38, 115 (2013). 23454933 10.1364/OL.38.000115

[b17] Shirai T. & Friberg A. T. Intensity-interferometric spectral-domain optical coherence tomography with dispersion cancellation. J. Opt. Soc. Am. A 31, 258 (2014). 10.1364/JOSAA.31.000258 24562023

[b18] Graf R. N. & Wax A. Temporal coherence and time-frequency distributions in spectroscopic optical coherence tomography. J. Opt. Soc. Am. A 24, 2186 (2007). 10.1364/josaa.24.002186 PMC2676227 17621322

[b19] Robles F., Graf R. N. & Wax A. Dual window method for processing spectroscopic optical coherence tomography signals with simultaneously high spectral and temporal resolution. Opt. Express 17, 6799 (2011). 19365509 10.1364/oe.17.006799 PMC2834290

